# Brain morphological changes in hypokinetic dysarthria of Parkinson's disease and use of machine learning to predict severity

**DOI:** 10.1111/cns.13304

**Published:** 2020-03-20

**Authors:** Yingchuan Chen, Guanyu Zhu, Defeng Liu, Yuye Liu, Tianshuo Yuan, Xin Zhang, Yin Jiang, Tingting Du, Jianguo Zhang

**Affiliations:** ^1^ Department of Neurosurgery Beijing Tiantan Hospital Capital Medical University Beijing China; ^2^ Department of Functional Neurosurgery Beijing Neurosurgical Institute Capital Medical University Beijing China; ^3^ Beijing Key Laboratory of Neurostimulation Beijing China

**Keywords:** brain morphology, hypokinetic dysarthria, machine learning, Parkinson's disease, structural magnetic resonance imaging

## Abstract

**Background:**

Up to 90% of patients with Parkinson's disease (PD) eventually develop the speech and voice disorder referred to as hypokinetic dysarthria (HD). However, the brain morphological changes associated with HD have not been investigated. Moreover, no reliable model for predicting the severity of HD based on neuroimaging has yet been developed.

**Methods:**

A total of 134 PD patients were included in this study and divided into a training set and a test set. All participants underwent a structural magnetic resonance imaging (MRI) scan and neuropsychological evaluation. Individual cortical thickness, subcortical structure, and white matter volume were extracted, and their association with HD severity was analyzed. After feature selection, a machine‐learning model was established using a support vector machine in the training set. The severity of HD was then predicted in the test set.

**Results:**

Atrophy of the right precentral cortex and the right fusiform gyrus was significantly associated with HD. No association was found between HD and volume of white matter or subcortical structures. Favorable and optimal performance of machine learning on HD severity prediction was achieved using feature selection, giving a correlation coefficient (*r*) of .7516 and a coefficient of determination (*R^2^*) of .5649 (*P* < .001).

**Conclusion:**

The brain morphological changes were associated with HD. Excellent prediction of the severity of HD was achieved using machine learning based on neuroimaging.

**Abbreviations**
DBS: deep brain stimulation
GLM: general linear model
HD: hypokinetic dysarthria
MDS‐UPDRS: Movement Disorder Society‐Sponsored Revision of the Unified Parkinson's Disease Rating Scale
MPRAGE: magnetization‐prepared rapid acquisition gradient echo
MRI: magnetic resonance imaging
MSE: mean square error
OFM1: orofacial motor cortex
PD: Parkinson's disease
ROIs: regions of interest
SVM: support vector machine
VHI: voice handicap index

## 1 INTRODUCTION

Parkinson's disease (PD) is the second most common progressive neurodegenerative disorder, with a prevalence of 1.6% among people of 65 years or older.^1^ The biological hallmark of PD is deposition of phosphorylated α‐synuclein in Lewy bodies and Lewy neurites. The Lewy body pathology contributes to neuronal loss in the pars compacta of the substantia nigra and is believed to spread from the brainstem to subcortical and cortical regions, eventually contributing to the motor and nonmotor symptoms of PD.^2^

Up to 90% of patients with PD eventually develop speech and voice disorders, referred to as hypokinetic dysarthria (HD).^3^ HD is manifested in all dimensions of human speech and voice production, specifically in the areas of articulation, phonation, prosody, speech fluency, and faciokinesis. HD is characterized by rigidity and bradykinesia, together with reduced muscular control of the larynx, articulatory organs, and other physiological support mechanisms of human speech production. Since self‐monitoring of speech is abnormal in PD, HD has a serious impact on the quality of life of PD patients.

Many researches have focused on the mechanism of HD in PD. An earlier study found that dysarthrophonia in PD patients is associated with functional anomalies in the basal ganglia, orofacial motor cortex (OFM1, part of precentral cortex), and cerebellum, together with increased recruitment of premotor and prefrontal cortices during speech production.^3^ Pinto et al used functional magnetic resonance imaging (fMRI) to compare limb vs speech movement activations in patients with PD. The study found aberrant and generally reduced neural activation during speech in patients with PD and also that additional effort and neural recruitment is necessary for patients with PD while performing dual‐motor tasks.^4^ On the other hand, Whitwell et al, using structural MRI on patients with progressive apraxia of speech, observed trends for fastest rates of decline in aphasia in patients with relatively small left, but preserved right, Broca area and precentral cortex. Bilateral reductions in lateral premotor cortex were associated with faster rates of decline of behavior.^5^ Abnormal function is known to be attributable to changes in brain morphology, but the morphological changes underlying HD are unclear and require investigation.

Evaluation of HD is tough, complicated, and time‐consuming, but plays a very important role in selection of medication, physical therapy, and other therapies.^6^, ^7^ For instance, deep brain stimulation (DBS) has been shown to be effective in relieving tremor, rigidity, and bradykinesia, but could aggravate and worsen HD symptoms.^8^ Thus, greater attention should be paid to applying DBS to patients with HD. So far, however, no reliable model has been developed to predict the severity of HD based on neuroimaging. Machine learning has been applied to disease diagnosis. Salvatore et al achieved an accuracy of 85.8% in individual PD diagnosis based on structural MRI using machine learning.^9^ Using machine learning, Shamir et al^10^ were also able to accurately predict 86% of motor improvement scores in patients who underwent DBS, based on neuroimaging and other features. Machine learning may, thus, potentially provide a solution for predicting the severity of HD although, so far, no model has been developed.

To address this challenge, here, we have investigated the association between HD and changes in brain morphology (including cortical thickness, white matter, and subcortical structure volume). After the complicated selection of appropriate features, we used a machine‐learning method that was able to predict the severity of HD.

## 2 METHODS AND MATERIALS

### 2.1 Subjects and neuropsychological measures

Patients at the Beijing Tiantan Hospital, Capital Medical University, China, between October 2018 and July 2019, were retrospectively included. All patients were diagnosed with idiopathic PD and met the UK Brain Bank criteria for a diagnosis of PD.^11^ Motor severity was defined using part III of the Movement Disorder Society‐Sponsored Revision of the Unified Parkinson's Disease Rating Scale (MDS‐UPDRS) and was assessed in the dopamine‐withdrawn state.^12^ The voice handicap index (VHI), which has been widely used to measure HD in PD patients, was used in the present study.^13^, ^14^ In total, 134 PD patients were included in the study and randomly assigned to Group A (n = 101) or Group B (n = 33).

### 2.2 MRI acquisition

Magnetic resonance imaging images of all subjects were acquired using 3.0 T MRI scanner (Philips Medical Systems) with 32‐channel head coil. The participants’ heads were immobilized accurately using head cushions. A whole‐head three‐dimensional sagittal T1‐weighted 3D magnetization‐prepared rapid acquisition gradient echo (MPRAGE) sequence was used (repetition time, 6.6 ms; echo time, 3.1 ms; flip angle, 8°; matrix size, 240 × 240; isotropic voxel, 1 × 1 × 1 mm^3^; number of slices, 196).

### 2.3 Imaging processing

All DICOM files were converted into NifTi format using SPM12 software (https://www.fil.ion.ucl.ac.uk/spm/software/spm12/), and the quality of the data (sharpness, whole‐head covering, orientation, etc) was carefully checked.

**Figure 1:**
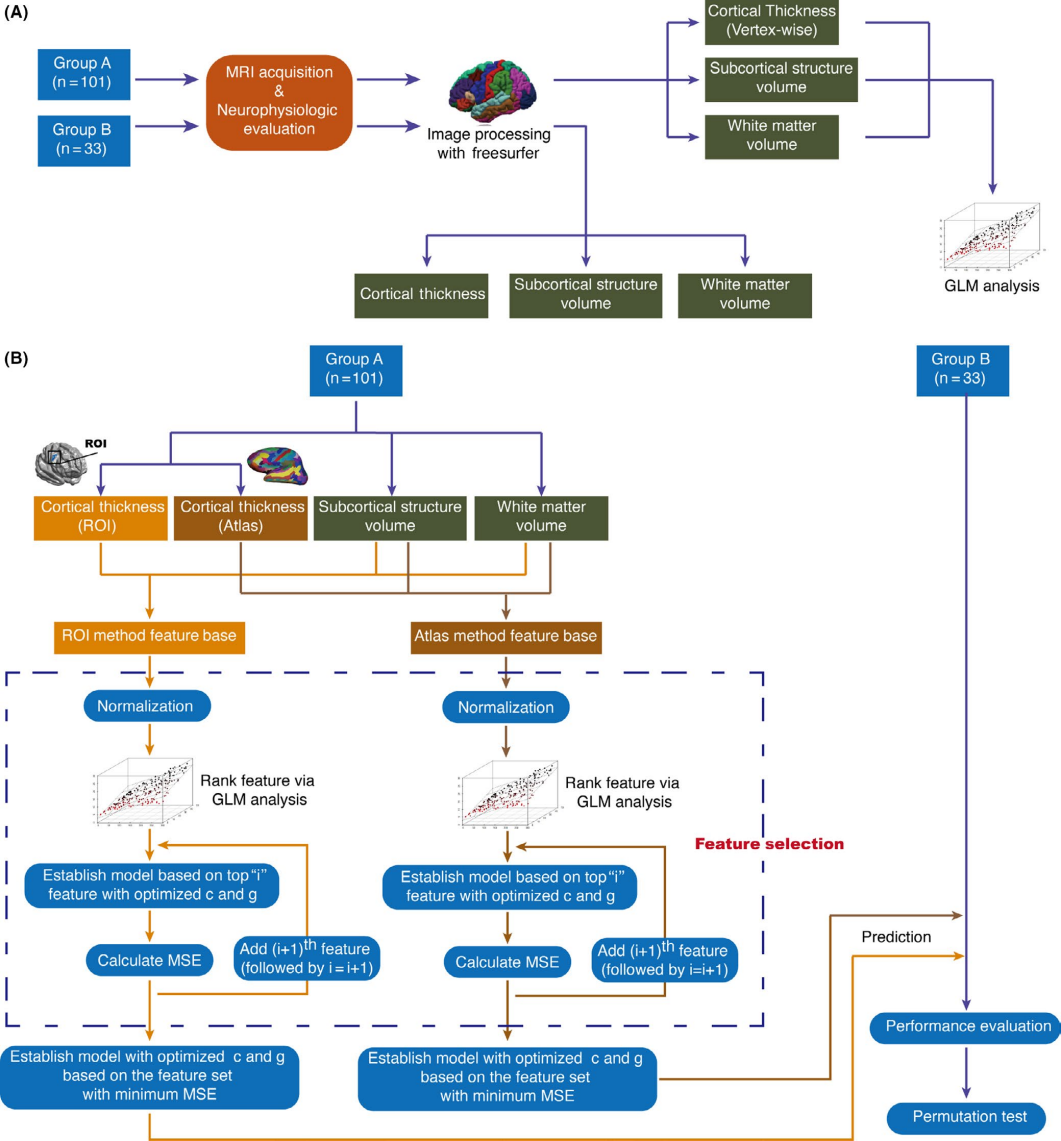
A, Flowchart showing data collection and processing. A total of 134 PD patients with comprehensive neuropsychological evaluation were included in this study. Structural MRI was performed on each subject, and all subjects were randomly assigned to Group A (n = 101) or Group B (n = 33). Association analysis between morphological changes (including cortical thickness, subcortical structure, and white matter volume) and hypokinetic dysarthria (HD) was conducted using the general linear model (GLM) in Group A. B, Flowchart showing prediction of HD by machine learning. In Group A (training set), cortical thickness (in terms of vertex‐wise analysis or atlas) and volumes of white matter and subcortical structures were considered to be features and included in the feature‐based regions of interest (ROIs) and atlas models. All features in each method were ranked based on normalized absolute values of β obtained using the GLM. The mean square error (MSE) of the model established by the top i feature was calculated, and the feature set with minimum MSE was selected and used to establish the final model. The model was then used to predict the severity of HD in the test set (Group B). The performance of the machine learning was evaluated followed by permutation test

Image analysis was performed using FreeSurfer software (version development, http://www.freesurfer.net). The technical details of primary cortical reconstruction and volumetric segmentation procedures have been previously described.^15^ Briefly, the processing included removal of nonbrain tissue using a hybrid watershed/surface deformation procedure, automated Talairach transformation, segmentation of the subcortical white matter and deep gray matter volumetric structures, intensity normalization, tessellation of the gray matter/white matter boundary, automated topology correction, and surface deformation following intensity gradients to optimally place the gray/white and gray/cerebrospinal fluid (CSF) borders at the location where the greatest shift in intensity defined the transition to the other tissue class. Cortical thickness was calculated as the closest distance from the gray/white matter boundary to the gray/CSF boundary at each vertex and finally registered to the FreeSurfer template.^16^ Adjustments were then made for differences in head size and volumes for each region (white matter and subcortical structure volume) were adjusted to intracranial volume (ICV), as previously described ^17^ (Figure 1A).

### 2.4 Feature selection and machine‐learning prediction of HD severity

Support vector machines (SVMs), one of the most important methods of machine learning, have been successfully used to solve data classification and regression problems because of their outstanding performance and small computation cost.^18^ In this study, the library for SVM (LIBSVM) (version 3.2, https://www.csie.ntu.edu.tw/~cjlin/libsvm/) package for MATLAB (version 2018b; MathWorks, Inc) was used.^19^ It should be mentioned that the random hyperparameter selection in the SVM implementation may cause poor robustness of the regressor; that is, the regression performance may be largely dependent on the randomly chosen initial conditions. Evolutionary computation algorithms could, however, be used to optimize the SVM hyperparameters in the hope of maximizing the effectiveness of the SVM.^20^ The genetic algorithm approach appears to be a good choice for the optimization.

To predict the severity of HD, Group A was set as the training set, Group B was set as the test set, and the data were normalized. The optimal SVM (with a radial basis function) hyperparameters were set when the mean square error (MSE) of cross‐validation (CV, 5‐fold) was minimum with the genetic algorithm (maximum generation, 200; population size, 20; cost range, 0, 100; gamma range, 0, 1000) in the training set. The model was then used to regress the test set.

Extraction of the feature of cortical thickness could be based on “vertex‐wise” analysis (known as the region of interest (ROI) method) or atlas segmentation (known as the atlas method). In the ROI method, ROIs were defined as the area that significantly correlated with HD (details shown in Section 2.5. Statistical analysis). The mean cortical thickness of each ROI, which was regarded as the feature of cortical thickness, was extracted from individuals and used for machine learning. In the atlas method, the brain was divided into different regions using the Destrieux atlas^21^ and the mean cortical thickness of each region was calculated and regarded as the feature of cortical thickness. The volumes of subcortical structure (“aseg.stats” file) and white matter (“wmparc.stats” file) were regarded as the features of subcortical structure and white matter, respectively, and were also included in the feature base of the ROI method and the atlas method.

To improve the performance of machine learning, feature selection was conducted before carrying out machine learning. This is important because some features are less sensitive, irrelevant, or redundant for classification, compared with others. Because of differences in the dimensions of feature values, and in order to make values of beta (β, the slope of the general linear model (GLM)) comparable, the values of different features were normalized via z‐scores, which is a common normalization method.^22^ The normalized β value of each feature was calculated based on the GLM: $y_{VHI\;\;score}=a_{i}+\beta_{i}\times X_{i}+\beta_{\mathrm{age}}\times X_{\mathrm{age}}+\beta_{\mathrm{sex}}\times X_{\mathrm{sex}}+\varepsilon_{i}$ ($y_{VHI\;\;score}$, VHI score; $a_{i}$, intercept of *i* ^th^ feature; $\beta_{i}$, slope of *i* ^th^ brain feature; $X_{i}$, value of *i* ^th^ brain feature (such as volume and thickness);$\beta_{\mathrm{age}}$, slope of age; $X_{\mathrm{age}}$, age; $\beta_{\mathrm{sex}}$, slope of sex;$X_{\mathrm{sex}}$, sex; $\varepsilon_{i}$, residual error of *i* ^th^ feature; computed independently for each feature, without considering the correlation with other features). The absolute values of each normalized $\beta_{i}$ were then ranked in descending order. The larger the absolute value of the normalized value of $\beta_{i}$, the more relevant the feature is to classification. An earlier study used the Pearson correlation coefficient to assess relevance,^23^ but cortical thickness and structural volume could be affected by aging.^24^ Therefore, only $\beta_{i}$ in the GLM represents relevance between brain structure and VHI, without being affected by aging and sex. Firstly, basic information (age and sex) and the brain feature with the largest $\beta_{i}$ were included in the SVM. Secondly, basic information and the two brain features with the largest values of $\beta_{i}$ were included in the model. This procedure was repeated until all brain features were included in the model. The optimal brain feature set was defined as that with which the model achieved the minimum MSE, using the feature set across all the models. The model established using these features was then used for predicting the test set. It should be emphasized that the feature selection procedure was performed only in Group A (the training set), which could eliminate the effect of selection bias on machine learning (Figure 1B).

### 2.5 Statistical analysis

Data are expressed as mean ± standard deviation. Clinical data and the basic information of Group A and Group B were analyzed using two‐sample t tests and chi‐squared tests.

In Group A, to evaluate cortical thickness, a surface‐based Gaussian smoothing kernel with full‐width half‐maximum of 10 mm was used before analysis, as previously described.^25^ Correlations between cortical thickness and VHI scores were modeled vertex‐wise. The offset and slope, which are subject‐independent regression coefficients, were estimated separately for each vertex using a GLM, while controlling for age and sex. Correlation coefficients were calculated from the slope and mapped onto the surface. The GLM was also used to measure correlations between white matter, subcortical structure volume, and VHI scores. The false discovery rate (FDR) method was used to control for multiple comparisons of different regions. In this step, no normalization method was involved.

The brain areas in which cortical thickness was significantly correlated with VHI score were set as ROIs. Evaluation indices of the SVM model, including the Pearson correlation coefficient (*r*) and coefficient of determination (*R^2^*), were computed as in previous studies.^26^ Permutation tests (2000 permutations) of *R^2^* were performed to further evaluate the performance of machine learning, based on the previous study ^27^ (Method S1).

Statistical analysis was carried out and plotted using MATLAB. A conventional *P* < .05 per comparison threshold was adopted.

## 3 RESULTS

### 3.1 Participant characteristics

A total of 134 patients with PD were included in this study and divided into Group A and Group B. There were no differences in age, sex, MDS‐UPDRS III, and VHI scores between the two groups. Detail information is summarized in Table 1.

**Table 1:** Participant characteristics

	Group A	Group B	*P* value
Number of patients	101	33	—
Age (years)	61.96 ± 9.06	61.03 ± 10.85	.6274
Sex (number of male/female)	56/45	21/12	.4087
MDS‐UPDRS III score	50.51 ± 17.62	51.18 ± 16.19	.8477
VHI score	25.52 ± 21.42	24.36 ± 20.50	.7852

Abbreviations: MDS‐UPDRS III, part III of Movement Disorder Society‐Sponsored Revision of the Unified Parkinson's Disease Rating Scale; VHI, voice handicap index.

### 3.2 Association between regional cortical thickness and severity of HD

**Figure 2:**
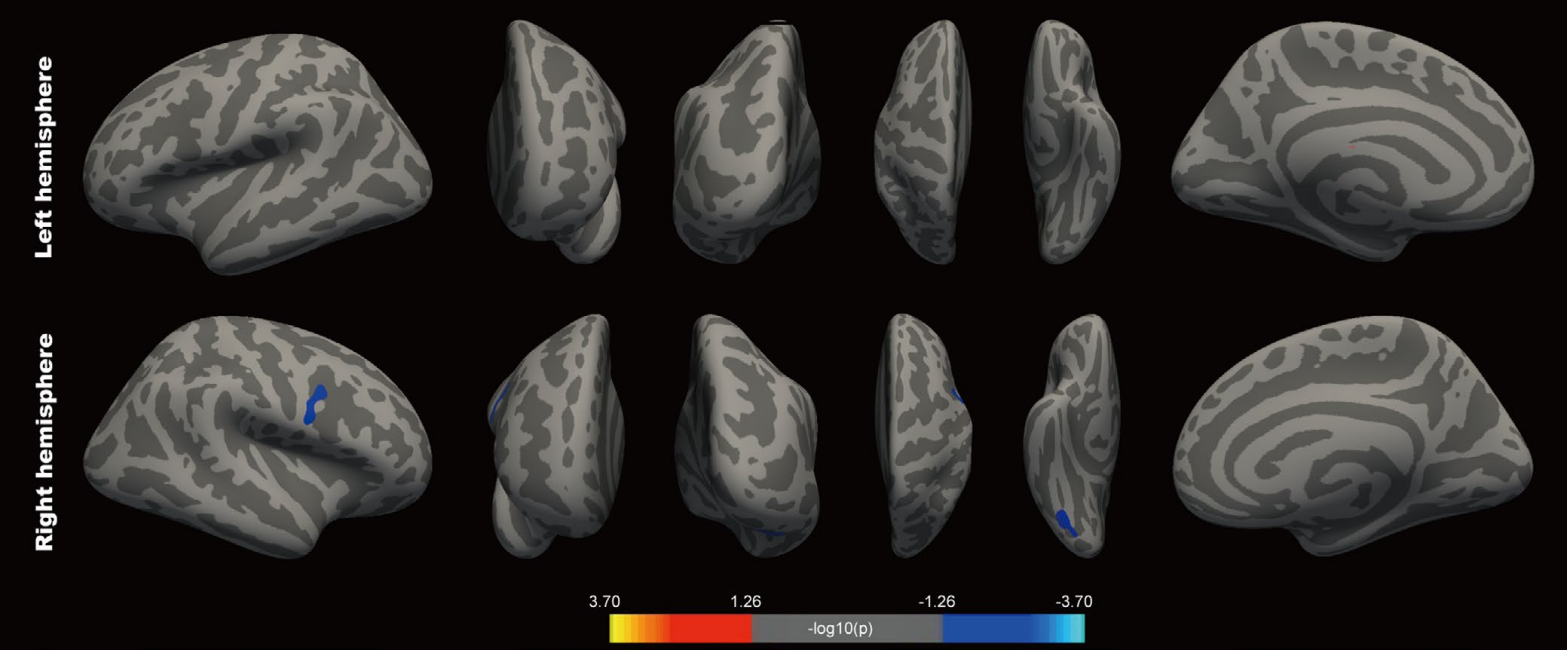
Vertex‐wise correlations between cortical thickness and severity of hypokinetic dysarthria (HD). Right precentral cortex and fusiform gyrus atrophy were associated with HD. Scale bar: cool color, negative correlation; warm color, positive correlation

To evaluate the association between cortical thickness and HD, a vertex‐wise analysis was carried out using the GLM as described above, in order to eliminate the effect of age and sex. HD was significantly negatively correlated with right precentral and fusiform cortical thickness (all *P* < .05) (Figure 2). The cluster size and MNI coordinates of these areas are summarized in Table 2.

**Table 2:** Significant cortical thickness associated with HD

	Cortical area	Cluster size (mm^2^)	Cluster‐wise *P* value	MNI coordinates (mm)		
				x	y	z
ROI 1	Right precentral	225.5	.0271*	57.2	6.0	22.3
ROI 2	Right fusiform	217.8	.0329*	35.9	−68.5	−13.4

Monte Carlo simulation.

Abbreviations: HD, hypokinetic dysarthria; ROI, region of interest.

^*^ *P* < .05.

### 3.3 Volumetry of subcortical structures and white matter

An earlier study confirmed a significant positive correlation between age and atrophy of subcortical structures and white matter impairment.^26^ The GLM, instead of the Pearson correlation, was therefore used to analyze the correlation between volume and HD. In this study, there was no obvious correlation between the volume of subcortical structures and HD (all *P* > .05). Similar results were obtained in volumetry studies of white matter (all *P* > .05).

### 3.4 Favorable performance of machine learning in predicting HD

**Figure 3:**
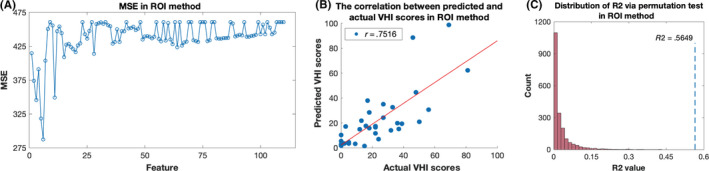
Feature selection and performance of machine learning in ROI method. A, Mean square error (MSE) of each feature set in the ROI method. The minimum MSE was 287.5 when the first six features (according to absolute value of $\beta_{i}$) were used in the training set via feature selection. B, Correlation between actual and predictive VHI scores. Favorable and significant results were achieved with an *r* value of .7516 and an *R^2^* value of .5649, indicating that this model can predict severity of hypokinetic dysarthria. C, Distribution of *R^2^* via permutation test in ROI method. A significant *P* value (*P* < .001) was achieved via permutation test

As described above, two feature bases were calculated and included in this study. For the ROI method, a total of 110 features were regarded as candidate features. The minimum MSE was 287.5 when the first six features (according to the weight of $\beta_{i}$) were included in the training set via feature selection. These were cortical thickness: ROI 1 and ROI 2; subcortical structure volume: left accumbens and right accumbens; and white matter volume: right medial orbitofrontal and right superior frontal white matter (Figure 3A and Table 3). The SVM regression model was established using these features (basic information (sex and age) and the six candidate features) and optimized hyperparameters (cost (c) and gamma (g)). Favorable and significant results were achieved, with the *r* value of .7516 and *R^2^* value of .5649. The permutation test confirmed that the result of the machine learning was significant, and the scatter plot showed an excellent correlation between actual and predicted VHI scores (Figure 3B,C and Table 3).

**Table 3:** Performance of machine learning in predicting VHI score

	ROI method	Atlas method
Features of brain morphology	Cortical thickness: ROI 1, ROI 2	Subcortical structure volume: left accumbens
	Subcortical structure volume: left accumbens, right accumbens	White matter volume: right superior frontal
	White matter volume: right medial orbitofrontal, right superior frontal	
*r*	.7516	.2721
*P* value of *r*	.0000**	.1255
*R^2^*	.5649	.0741
*P* value of permutation test (for *R^2^*)	.0000**	.0465*

Note

*r*, Pearson correlation coefficient; *R^2^*, coefficient of determination.

^*^ *P* < .05.

^**^ *P* < .001.

**Figure 4:**
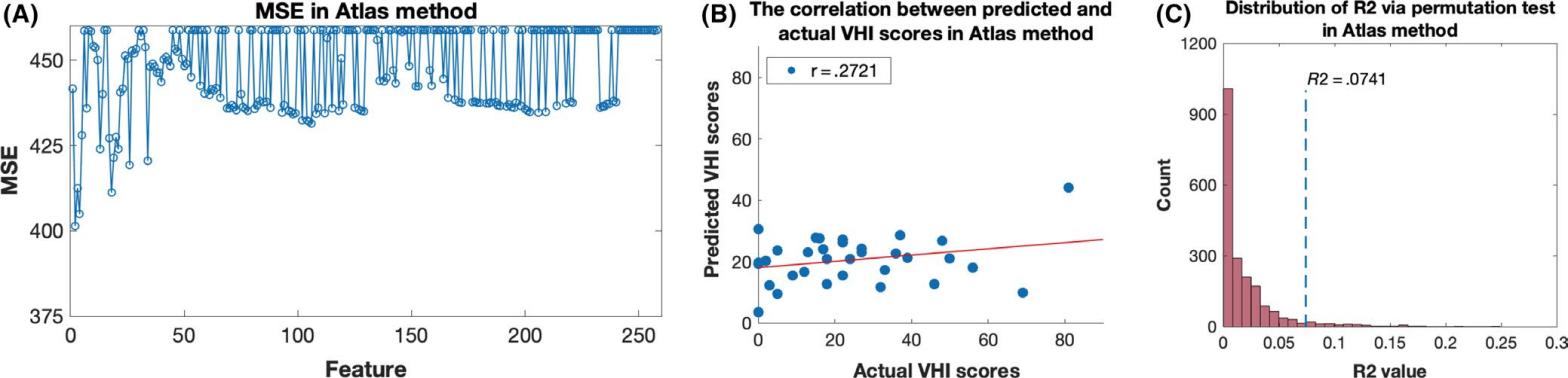
Feature selection and performance of machine learning in atlas method. (A) Mean square error (MSE) of each feature set in atlas method. The minimum MSE was 401.5 when the first two features (according to absolute value of $\beta_{i}$) were used in the training set via feature selection. B, Correlation between actual and predictive VHI scores. Acceptable results were achieved with an *r* value of .2721 and an *R^2^* value of .0741, which were lower than the values of the ROI method. C, Distribution of *R^2^* via permutation test in ROI method. A significant *P* value (*P* < .05) was achieved via permutation test

The performance of machine learning based on the atlas method was also evaluated. A total of 258 features were regarded as candidate features. During feature selection, a minimum MSE of 401.5 was achieved with two features (subcortical structure volume: left accumbens; white matter volume: right superior frontal white mater) included in the SVM model (Figure 4A and Table 3). The hyperparameters of the model were optimized and used in the training set to establish the SVM model. Although the performance of machine learning based on the atlas method was significant, as confirmed by the permutation test, lower values of *r* (.2721) and *R^2^* (.0741) were obtained, compared with the ROI method. The predictive value of the model based on the atlas method was, therefore, less meaningful than that based on the ROI method (Figure 4B,C and Table 3).

## 4 DISCUSSION

Almost all of PD patients (up to 90%) eventually develop HD, which seriously decreases their quality of life. Previous studies showed that functional abnormalities in multiple brain regions were associated with HD.^3^ The morphological brain changes in PD patients that are associated with HD, however, remain unclear. Evaluation of HD is also really complicated and time‐consuming, and a reliable predictive model of this symptom is still lacking. Here, we investigated the association between severity of HD and cortical thickness, subcortical structure, and volume of white matter. Using machine learning, we went on to develop a predictive model of HD based on brain morphology and obtained favorable predictive values.

In general, HD can be analyzed in two ways: (1) perceptive analysis using a scale based on speech and voice or (2) acoustic analysis of speech waveforms. In terms of perceptive analysis of HD in PD patients, some neuroscience‐oriented research has used the UPDRS III: motor examination, item 18, for the evaluation of speech production, rated on a 0‐4 scale. This is, however, only a screening tool and provides an insufficient detailed measure of HD.^28^ VHI, which consists of functional, physical, and emotional subscales, rated from 0 (never) to 4 (always), with higher scores indicating increasing severity of voice disability, has been widely adopted by many researchers.^13^ Significant correlation has been found between VHI scores and the patients' self‐rated dysphonic severity.^29^ Acoustic analysis could be affected by different medication states and may not reflect the recent status. Additionally, special equipment and software are needed for acoustic analysis.^30^ VHI was, therefore, used to evaluate HD, as in previous studies.^31^, ^32^ In this study, we evaluated the severity of HD, rather than classifying PD patients into those suffering HD and those not suffering HD because of the lack of a uniform standard (cut value).^33^, ^34^ Furthermore, some patients did not reach the criteria of HD, but suffered subclinical HD. There is no doubt that the VHI score reflects the severity of HD in PD patients.^35^ The previous study report that association between voice and motor disabilities was observed,^36^ similarly, our results indicated a significant correlation between HD (VHI score) and motor impairment severity (MDS‐UPDRS III score) (*P* = .0059).

### 4.1 Association between morphological changes and HD

In this study, the severity of HD was found to be significantly positively associated with precentral and fusiform cortical atrophy. Furthermore, the area that was significantly associated with HD in the precentral cortex was mainly located on OFM1, according to the previous study and the atlas of the human brain.^37^

The HD‐related articulatory networks comprise both cortical and subcortical structures that include the anterior components of the dorsal language pathway (precentral cortex, inferior frontal gyrus, dorsolateral premotor cortex, supplementary motor area (SMA), and insula), as well as the basal ganglia, thalamus, and cerebellum.^28^ Planning and execution of speech depends on the integrity of cortico‐basal ganglia‐thalamo‐cortical loop, particularly the motor and associative cortico‐striatal loops. Primary and nonprimary motor areas connect to the posterior third of the striatum. The fiber connections are established with the external and internal pallidum, subthalamic nucleus, and ventrolateral thalamic nuclei. The ventrolateral thalamic nuclei project back to the primary and nonprimary cortical motor areas.^38^

In some studies, abnormal brain region connectivity and activity have been observed using fMRI. A significant association between levodopa‐induced changes in the OFM1‐SMA and connection strengths in the right caudate nucleus‐dorsolateral prefrontal cortex and medication‐induced changes in the acoustic parameters underlying control of pitch variation has been previously described.^7^ Arnold et al^3^ found that the connection between the right OFM1 and the superior temporal gyrus was correlated with parameters underlying modulation of voice intensity, regardless of medication status. $H_{2}^{15}$ O‐PET has been used to reveal neural correlates of HD and its treatment. One previous study confirmed that behavioral changes in PD patients brought about by Lee Silverman voice treatment were correlated with changes in regional cerebral blood flow within the right motor, prefrontal and temporal cortical regions, but not in the basal ganglia.^39^ Noninvasive brain stimulation of the precentral cortex with high‐frequency repetitive transcranial magnetic stimulation also led to increased speech rate and tongue movements, and improved voice quality and intensity in PD patients.^28^ The fusiform gyrus is closely associated with speech and speech perception. One study reported a patient with paroxysmal aphasia evoked by ictal epileptiform discharges localized to the fusiform gyrus, where a small brain tumor existed, and the patient also showed transient aphasia with electrical stimulation of foci.^40^ Another study found the anterior fusiform gyrus was modulated by voice identity only in late blind but not in matched sighted controls.^41^

In this study, no morphological changes were observed in white matter or subcortical structures. This may be because functional changes occur prior to observable morphological changes in PD patient with HD.

### 4.2 Machine learning for the prediction of HD severity

To ensure the reliability and precision of the results, feature selection was only performed within the training set, thus preventing the machine‐learning model from inspecting the data of the test set during the process of model establishment. As mentioned above, two ROIs with significant *P* values were obtained. Other features without significant *P* values, including volume of white matter and subcortical structure, may also be associated with HD. In other words, these features could still be candidate features. To further illustrate this point, the performance of machine learning based on two ROIs and basic information was evaluated. An *r* value of .2263 and an *R^2^* value of .0512 indicated a lower performance than that of the ROI method (Figure S1). Comparing the performance of the ROI method and the atlas method, the model based on ROIs was found to have higher predictive value. This may be because the atrophied areas that are significantly associated with HD are focal. In other words, only a focal region (OFM1) of the right precentral cortex was found to be associated with HD, whereas in the atlas method, the whole right precentral cortex was considered to be one region. Using the mean thickness of the whole right precentral cortex may, therefore, not well represent the morphological changes in HD and may lower the performance of machine learning.

Machine learning has been widely used in PD, especially in disease diagnosis. A variety of studies have attempted to use machine learning to diagnosis PD based on one or more neuroimaging techniques, including structural MRI, diffusion tensor imaging, fMRI, and PET, with an accuracy of 65.7%‐86%.^42^, ^43^ Prediction of the severity of symptoms, however, appears to be a huge challenge. Few studies have attempted to predict motor performance by machine learning based on neuroimaging. One previous study reported *r* values of .35 and .45 in predicting UPDRS III improvement after DBS.^44^ Relatively high values of *r* (.7516) and *R^2^* (.5649) were achieved in this study, indicating a favorable performance in predicting the severity of HD, which could be used clinically in the evaluation of HD and selection of therapeutic method. Larger, prospective, and multiple center study could be conducted in the future in order to extend suitability.

## 5 CONCLUSION

The morphological changes associated with the severity of HD have not been previously described. In this study, it was found that atrophy of the right precentral cortex, specifically the right OFM1, and the fusiform gyrus was associated with HD in PD patients. No association was found between HD and volume of white matter or subcortical structures. Machine learning based on structural MRI performed well in predicting the severity of HD (*r* = .7516 and *R^2^* = .5649) and has a potential clinical value in the future.

## CONFLICT OF INTEREST

The authors declare no conflict of interest.

## Supporting information

Figure S1

Method S1
