# Supplementary figures and images for: Brain morphological changes in hypokinetic dysarthria of Parkinson's disease and use of machine learning to predict severity
Source: CNS Neurosci Ther. 2020 Mar 20;26(7):711–9. doi: 10.1111/cns.13304 (PMC7298984; doi:10.1111/cns.13304)

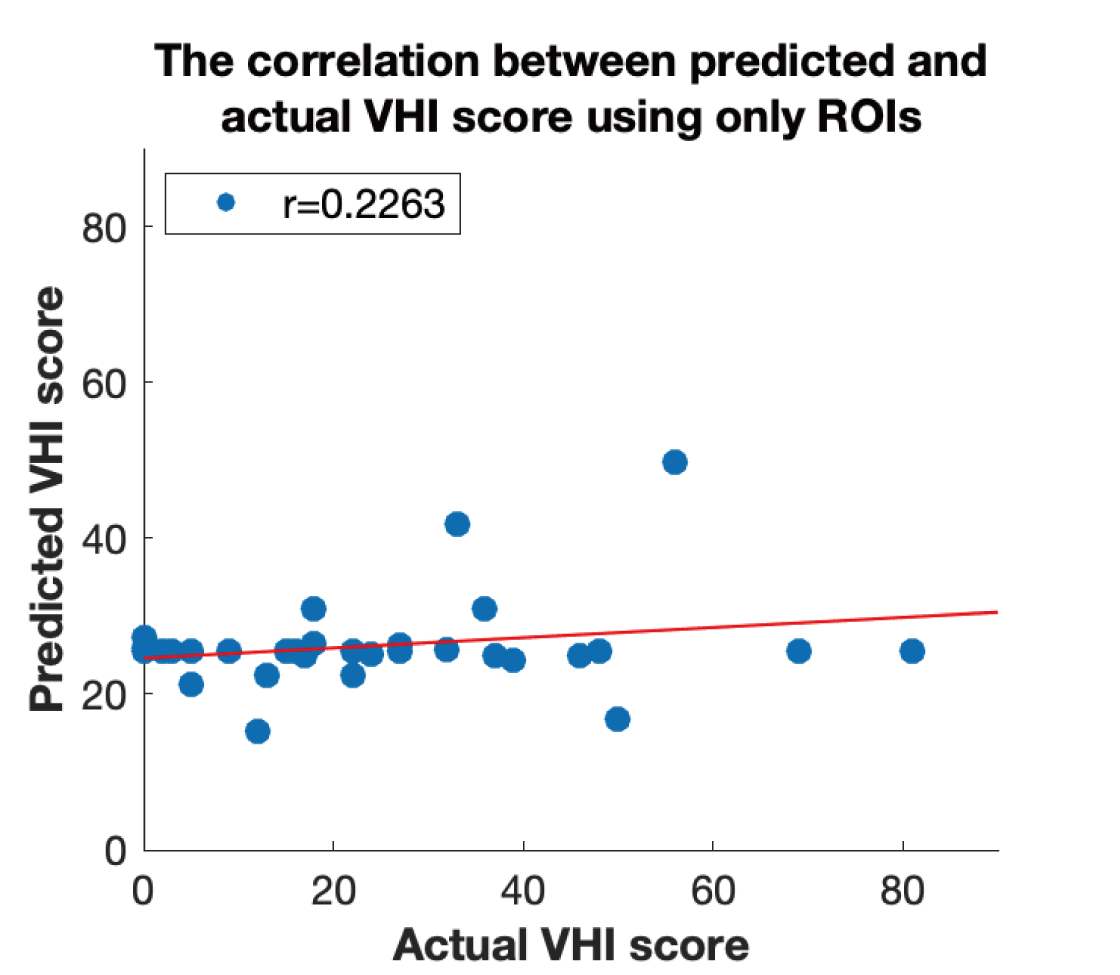

Supplement: Supplementary file 1 — Figure S1 [file CNS-26-711-s001.tif]
