# Supplementary material for: Brain morphological changes in hypokinetic dysarthria of Parkinson's disease and use of machine learning to predict severity
Source: CNS Neurosci Ther. 2020 Mar 20;26(7):711–9. doi: 10.1111/cns.13304 (PMC7298984; doi:10.1111/cns.13304)
Supplement: Supplementary file 2 — Method S1 [file CNS-26-711-s002.docx]

**Supplementary Method**

**2.5. Statistical analysis**

In this study, we permuted the labels 2000 times. Each time randomly assigning labels to each image and repeated the feature selection, hyperparameter optimization and prediction et.al. We then counted the number of times the R^2^ for the permuted labels were higher than the ones obtained for the real labels. Dividing this number by 2000, we derived a P value for the regression.
